# Microwave Ablation for Colorectal Liver Metastases: A Systematic Review and Pooled Oncological Analyses

**DOI:** 10.3390/cancers14051305

**Published:** 2022-03-03

**Authors:** Antonio Mimmo, Francesca Pegoraro, Rami Rhaiem, Roberto Montalti, Alix Donadieu, Ahmad Tashkandi, Abdul Rahman Al-Sadairi, Reza Kianmanesh, Tullio Piardi

**Affiliations:** 1 Department of Hepatobiliary, Pancreatic and Digestive Oncological Surgery, Reims Medical Faculty, Robert Debré University Hospital, University of Reims Champagne-Ardenne, Rue du Général Koenig, 51100 Reims, France; francesca.pego@libero.it (F.P.); rrhaiem@chu-reims.fr (R.R.); alixdonadieu@yahoo.fr (A.D.); atashkandi@chu-reims.fr (A.T.); alsideiriab@gmail.com (A.R.A.-S.); rkianmanesh@chu-reims.fr (R.K.); 2 Division of Hepato-Bilio-Pancreatic, Minimally Invasive and Robotic Surgery, Department of Clinical Medicine and Surgery, Federico II University Hospital, Via S. Pansini 5, 80131 Naples, Italy; roberto.montalti@unina.it; 3 Research Unit Ea3797 VieFra, Department of Hepatobiliary, Pancreatic and Digestive Oncological Surgery, Reims Medical Faculty, Robert Debré University Hospital, University of Reims Champagne-Ardenne, Rue du Général Koenig, 51100 Reims, France; tpiardi@chu-reims.fr

**Keywords:** colorectal liver metastasis, microwave ablation, liver resection

## Abstract

**Simple Summary:**

Liver resection for colorectal liver metastases (CRLM) represents the best curative option; however, few patients are candidates for surgery. Microwave ablation (MWA) can be a valid alternative in selected patients. This systematic review reports the oncological results of MWA for CRLM. The literature available on the Web was analyzed for reports concerning MWA for resectable CRLM, published before January 2021. Finally, 12 papers concerning MWA complications, recurrence-free (RF) cases, patients free from local recurrence (FFLR), and overall survival rates (OS) were selected. Global RF rates at 1, 3, and 5 years were 65.1%, 44.6%, and 34.3%, respectively. Global FFLR at 3, 6, and 12 months were 96.3%, 89.6%, and 83.7%, respectively. Global OS rates at 1, 3, and 5 years were 86.7%, 59.6%, and 44.8%, respectively. A better FFLR was achieved with an MWA surgical approach at 3, 6, and 12 months, with 97.1%, 92.7%, and 88.6%, respectively. Surgical MWA for CRLM smaller than 3 cm was a safe and valid option. MWA can be entered as part of the flowchart decision of CRLM curative treatment, especially for use in the parenchyma-sparing strategy and as a complement to surgery.

**Abstract:**

(1) Background: colorectal liver metastases (CRLM) are the most common extra-lymphatic metastases in colorectal cancer; however, few patients are fit for curative surgery. Microwave ablation (MWA) showed promising outcomes in this cohort of patients. This systematic review and pooled analysis aimed to analyze the oncological results of MWA for CRLM. (2) Methods: Following PRISMA guidelines, PubMed, Scopus, EMBASE, Google Scholar, Science Direct, and the Wiley Online Library databases were searched for reports published before January 2021. We included papers assessing MWA, treating resectable CRLM with curative intention. We evaluated the reported MWA-related complications and oncological outcomes as being recurrence-free (RF), free from local recurrence (FFLR), and overall survival rates (OS). (3) Results: Twelve out of 4822 papers (395 patients) were finally included. Global RF rates at 1, 3, and 5 years were 65.1%, 44.6%, and 34.3%, respectively. Global FFLR rates at 3, 6, and 12 months were 96.3%, 89.6%, and 83.7%, respectively. Global OS at 1, 3, and 5 years were 86.7%, 59.6%, and 44.8%, respectively. A better FFLR was reached using the MWA surgical approach at 3, 6, and 12 months, with reported rates of 97.1%, 92.7%, and 88.6%, respectively. (4) Conclusions: Surgical MWA treatment for CRLM smaller than 3 cm is a safe and valid option. This approach can be safely included for selected patients in the curative intent approaches to treating CRLM.

## 1. Introduction

Colorectal cancer (CRC) is the third most common cancer worldwide, with 25–35% of patients presenting with or developing colorectal liver metastases (CRLM). Almost 20% of patients present synchronous CRLM and 10–15% of patients, metachronous CRLM [1,2]. Liver resection is the current gold standard for the curative-intent treatment of CRCLM, leading to a 5-year overall survival (OS) rate of 31–58% [3]. Unfortunately, only 15–25% of patients are suitable for oncological surgery, mainly due to patient factors (age, medical condition) and/or tumor factors (size, number, and localization) [4]. Modern chemotherapy (CHT) alone has improved the OS by 15–20 months [5]. To achieve better oncological results, local therapies, such as thermal ablation and external beam radiation (SBRT), with or without surgery have been developed. These techniques are aimed at optimizing the outcome by maximizing parenchymal preservation and minimizing the local recurrence [6,7]. In these settings, ablation techniques offer an advantage for surgery, treating deep lesions more easily [6,7]. The long-term reported results after radiofrequency ablation (RFA) for CRLM are promising; this technique represents the most commonly used modality, before microwave treatment and cryotherapy [8]. Recurrence rates from RFA are approximately three times lower than those for cryotherapy, but they are approximately three times higher compared to resection when used as a first-line treatment in patients with resectable disease [9,10,11]. RFA is safe and effective, both in percutaneous and surgical approaches, although its limitations include increased impedance as temperatures reach 100 degrees Celsius, a small zone of active heating, and decreased effectiveness with charring. The results of RFA in terms of outcome and safety have been extensively demonstrated [12,13,14]. RFA is less useful for the treatment of lesions near to large blood vessels because of the “heat-sink effect”, where the flowing blood carries heat away, bringing higher local recurrence rates. The principle of microwave ablation (MWA) is the tissue coagulation necrosis caused by the oscillation of polar molecules, with non-reliance on electrical conductivity; this procedure is less affected by the presence of blood vessels [15,16]. This technique also remains effective in temperatures above 100 degrees Celsius, allowing for the use of multiple antennae providing a potentially larger ablation zone, with a shorter procedure time [17,18]. In the past few years, MWA has established a larger place in the treatment of CRLM because of the previously cited reasons [19]. Interestingly, some authors suggested increasing the ablation zone by reducing the surrounding blood inflow during intraoperative MWA, by clamping the hepatic pedicle [20,21]. Microwave (MW) energy is more difficult to distribute than radiofrequency (RF) energy. MW energy is carried in wavelengths, which are more cumbersome than the small wires used to feed energy to RF electrodes and are prone to heating up when carrying a large amount of power. Consequently, MWA appears less feasible than RFA in the treatment of high-risk-located and subcapsular nodules. In addition, MWA is more expensive than RFA [22]. When comparing RFA and MWA, patients who underwent MWA had lower ablation-site recurrence rates (6% vs. 20%), and the 2-year control rate was significantly lower (7% vs. 18%) [23]. The existing literature on the outcomes following MWA for CRLM remains heterogeneous and sparse. This systematic review aims to report the results of the current state of evidence for CRLM treated by MWA, evaluating the oncological outcome in terms of overall survival, recurrence-free survival, and local recurrence-free survival, with all reporting enhanced by the benefits of pooled analysis.

## 2. Materials and Methods

The literature search methods, selection criteria, data collection, outcome measurements, and statistical analysis methods were selected according to the preferred items for systematic reviews and meta-analysis (PRISMA) statement [24]. No institutional review board or ethics committee approval was required.

### 2.1. Search Strategy

An online search of the PubMed, Scopus, EMBASE, Google Scholar, Science Direct, and the Wiley Online Library databases was performed to identify all studies on the treatment of CRLM through MWA ablation. Additional gray literature was searched using the POPLINE and SIGLE database to include doctoral dissertations, theses, and publications not found in the main databases. Additionally, we searched the Cochrane, mRCT, and clinicaltrials.gov databases to scan the ongoing or completed clinical trials on the matter.

When studies with overlapping cohorts were encountered, the report with a higher number of subjects or with the longest follow-up period was selected.

The systematic search of the literature was performed without publication-period restriction and was completed on 1 January 2021, including only those reports written in English.

A medical subject headings (MeSH) search was conducted for PubMed, Scopus, EMBASE, and Cochrane databases, applying the following terms: “((microwave) OR (MW) OR (MWA)) AND ((liver) OR (hepatic)) AND (metastases) AND ((colorectal) OR (colon) OR (colo-rectal))”.

To include the highest possible number of papers, the other databases were scanned using “Colorectal Liver Metastases Microwave” as a query.

References cited in the relevant papers were also examined to include all potentially noteworthy reports.

### 2.2. Selection Criteria

Randomized controlled trials (RCT) were studied, as well as observational studies with or without control arms that enrolled patients with CRLM treated via MWA.

**Figure 1:**
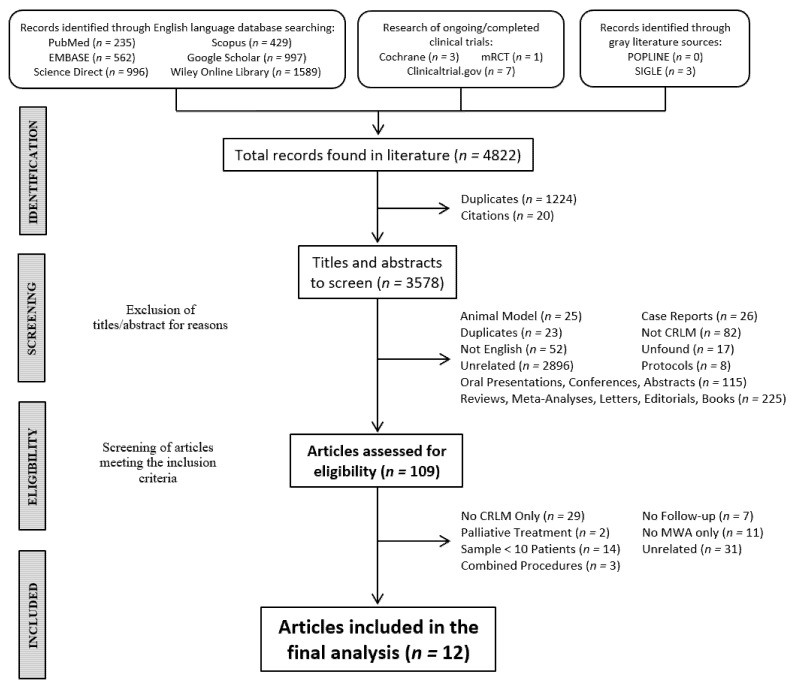
Flowchart showing the selection process of the included studies. Abbreviations: colorectal liver metastases (CRLM), microwave ablation (MWA).

Two reviewers (A.M. and F.P.) independently reviewed the results obtained by a literature search in a two-step method (Figure 1). After duplicates and citations were removed, the first step consisted of screening the titles and abstracts to determine their eligibility and relevance to the topic. During this phase, case reports, oral presentations, conferences, abstracts without a corresponding full text, reviews, meta-analyses, letters, editorials, books, non-English articles, reports describing study protocols, animal-model studies, and unrelated articles were excluded. In case of doubts or ambiguous abstracts, those studies were selected for full-text screening.

During the second step, a full-text screening of articles meeting the specific inclusion criteria was made by the same reviewers. Studies were excluded when they did not include MWA as a treatment, had a sample of fewer than 10 patients, used MWA as a palliative treatment, did not report a sufficient follow-up, combined MWA and other treatments on the same subjects, or were unrelated, as well as those studies in which the patients affected by CRLM and/or treated with MWA could not be correctly isolated from those with other liver metastases and/or were treated with other procedures (i.e., RFA). We excluded all reports with a sufficient follow-up time that failed to report our outcomes of interest. Any disagreement was jointly resolved by the authors and reviewed by two expert surgeons (R.K., T.P.).

The exclusion and inclusion criteria are summarized in Table 1.

### 2.3. Data Extraction and Outcomes of Interest

Once the articles meeting the specific inclusion criteria were identified, a series of datasets were extracted independently by the two reviewers. These included: study type and design, patients’ characteristics, the total number of MWA performed, mean/median lesion dimensions, the number of lesions per patient, synchronous/metachronous lesions, treatment at recurrence, operation time, length of hospital stay, complications, follow-up time, and the items defining the inclusion criteria (see below). All the selected articles evaluated the postoperative complications using the Clavien–Dindo Score (CDs) [25]. Additional data, such as neoadjuvant and adjuvant treatments, specific postoperative complications, and types of MWA device and needle tip were registered as well.

Subsequently, and upon existing data, we stratified for lesions according to diameter (<30 mm vs. >30 mm), for percutaneous/surgical treatment procedures, and for the type of surgical approach (open, vs. laparoscopic, robotic) in the latter group.

The following primary outcome was extracted: rates of patients free from local recurrence (FFLR, at 3, 6, and 12 months at least). The following secondary outcomes were extracted: recurrence-free rates (RF, at 3, 6, and 12 months, at least) and overall survival rates (OS, at 3, 6, and 12 months, at least). We defined the overall recurrence rate as the appearance of new lesions during postoperative follow-up, irrespective of their location. Local recurrence rate was defined as a postoperative relapse occurring specifically on the ablated liver lesions.

Additional outcomes included the evaluation of recurrence-pattern patients with hepatic and extra-hepatic progression.

### 2.4. Quality Assessment

The quality of all included studies was assessed independently by A.M. and F.P. using the Newcastle-Ottawa scale (NOS) of quality assessment [26]. Any disagreements were resolved in consensus.

### 2.5. Statistical Analysis

Data of interest were collected as presented in the original manuscripts or were calculated from the reported raw data whenever possible. Quantitative data were presented descriptively as mean and standard deviations (SD). When continuous data were presented as medians and range, the method developed by Hozo et al. was used [27]. When continuous data were presented as medians and interquartile range, we used the method developed by Wan et al. [28]. Categorical variables were summarized as frequencies and percentages with 95% confidence intervals (CIs).

We extracted the recurrence and survival outcomes from the Kaplan–Meier curves and/or explicit data, when available. Subgroup survival analysis on patients with lesions of <30 mm, treated percutaneously or surgically, was performed as well as outcomes after MWA under laparoscopic versus open procedures. Finally, grouped analysis incorporated papers providing data on the local recurrence rates.

## 3. Results

### 3.1. Literature Analysis

The literature search identified 4822 records. After excluding duplicates and citations, a pool of 3578 papers was screened. The first step of the literature analysis consisted of a screening of titles and abstracts to determine their eligibility and relevance to the topic: case reports, oral presentations, conferences, abstracts without a corresponding full text, reviews, meta-analyses, letters, editorials, books, non-English articles, reports describing study protocols, animal-model studies, and unrelated articles were excluded. After excluding 3469 papers, 109 articles were selected as being eligible for second-phase analysis.

The second step consisted of an analysis of the full text of articles meeting the specific inclusion criteria. Studies not including MWA as an exclusive treatment, with a sample of fewer than 10 patients, using MWA as a palliative treatment, not reporting the follow-up, combining MWA and other treatments on the same subjects, unrelated subjects, and those studies in which the patients who were affected by CRLM and/or treated with MWA could not be correctly isolated from those with other liver metastases and/or treated with other procedures (i.e., RFA) were excluded. Finally, 12 papers were included in the pooled analysis (Figure 1).

### 3.2. Study Characteristics

The general characteristics of these twelve papers are shown in Table 2, Table 3, Table 4 and Table 5 [20,29,30,31,32,33,34,35,36,37,38,39]. Eight studies (66.7%) were retrospective [29,30,32,34,36,37,38,39], and a total of 395 patients with CRLM were treated with MWA, 257 of whom were male (65%). The mean patients’ age was 58.9 ± 9 years. The mean lesion diameter was 17.6 ± 7.8 mm, of which 390 lesions (91.6%) were smaller than 30 mm. Percutaneous MWA treatment was performed in 108 (32.3%) of the patients, while 287 (85.9%) underwent a surgical procedure. Of the cases of surgical MWA, 34.7% were performed via the open approach and 47.5% were performed using a laparoscopic approach. Overall, 47.2% of patients presented synchronous CRLM. Regarding oncologic treatment, 70.5% and 90.4% of patients received neoadjuvant and adjuvant CHT, respectively. The type of MWA device, the MWA needle, the number of ablations per lesion, ablation time, and energy employed were reported on almost all papers; notably, there is a heterogeneity of protocols that appears to be specific for each Center (Table 4). The complications rate was 26.3%, as specified in Table 4. Serious complications (CDs ≥ 3) were reported in 8.4% of patients. The mean length of hospital stay was 5.43 days. Global survival analysis was reported in Table 5. The mean follow-up was 20.5 months (±9.6). The pooled data analysis on the oncological outcomes of MWA shows a global recurrence-free (RF) rate at 3 months, 6 months, and 1, 3, and 5 years of 95.5%, 89.5%, 65.1%, 44.6%, and 34.3%, respectively, resulting in a global RF of 37.1%. In total, the rates of patients free from local recurrence (FFLR) at 3, 6, and 12 months were 96.3%, 89.6%, and 83.7%, respectively. The overall recurrence rate was 41.36%. Intrahepatic progression during the follow-up appeared in 35.5% of patients, while 32.9% showed extra-hepatic progression. Global overall survival rates (OS) at 3 months, 6 months, and 1, 3, and 5 years were 99.3%, 97.3%, 86.7%, 59.6%, and 44.8%, respectively.

### 3.3. Subgroup Analysis

To better understand the outcomes of MWA-treated patients, we sorted them into sub-categories and analyzed the outcomes of these subgroups.

#### CRLM with a Diameter of ≤30 mm

**Figure 2:**
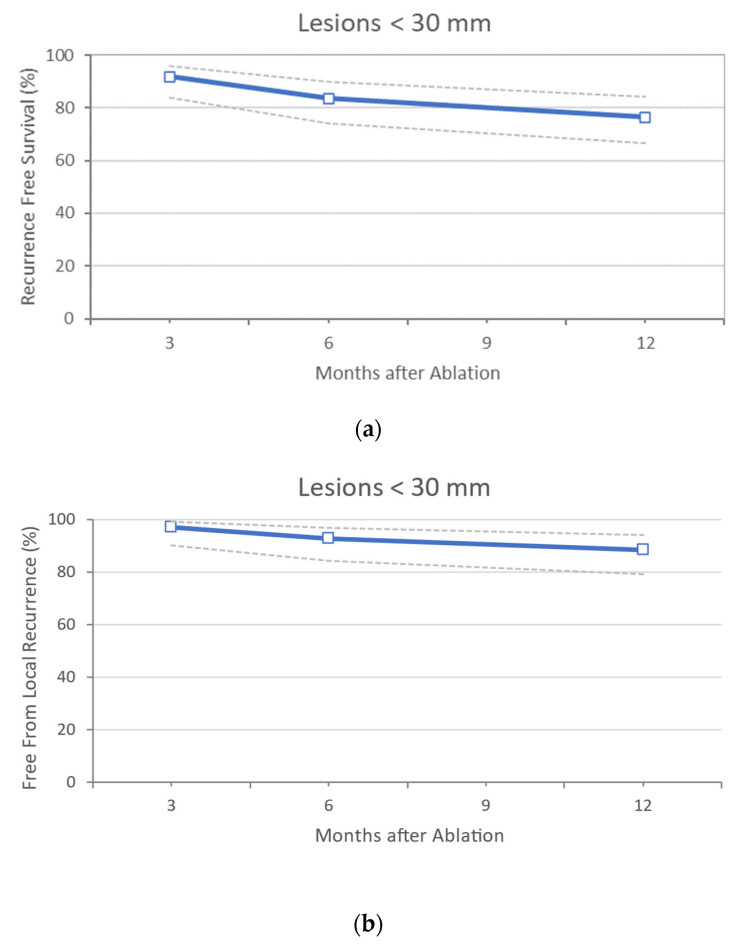
(**a**) Sub-analysis on studies comprising lesions <30 mm only: Recurrence Free Survival data. In dotted lines are indicated the 95% confidence intervals, (**b**) Sub-analysis on studies comprising lesions <30 mm only: Free from Local Recurrence outcome. In dotted lines are indicated the 95% confidence intervals.

Only 3 studies report an acceptable follow-up in this cohort subcategory [20,33,38] (Table 6 and Table 7). Eighty-five patients with 159 lesions of ≤30 mm were described, with a mean reported diameter of 20.39 mm (±2.9 mm). Two series performed surgical MWA [20,38] (82.4% of the total). Of these, 64.7% benefited from a laparoscopic approach. Multifocality (i.e., presence of more than one lesion) was present in 64.7% of patients, and most CRLM were metachronous (76.5%). All patients received neoadjuvant and adjuvant treatment. In this group of analyses, 16.5% of patients experienced complications, but none of these patients had CDs ≥ 3. The RF and FFLR at 3, 6, and 12 months were 91.8%, 83.5%, 76.5%, and 97.1%, 92.9%, and 88.6%, respectively, with hepatic progression of 32.4%. The overall recurrence was 28.2% (Figure 2a,b). The OS at 3, 6, and 12 months was 100%.

#### Surgical vs. Percutaneous MWA

Eight studies reported data concerning MWA conducted through a surgical approach [20,29,30,32,35,37,38,39] (Table 8 and Table 9). In this group of analyses, 602 lesions were treated in 287 patients, and the mean diameter of the lesions was 15.51 mm (±7.16 mm). The vast majority of surgically treated CRLM (96.9%) were smaller than 30 mm, and 57.8% of patients received a laparoscopic therapeutic approach. Most surgically treated MWA patients (72.6%) had more than one lesion, while 55.9% had synchronous CRLM. Complications after surgery were reported in 26.8% of patients, of which 8.5% were severe (CDs ≥ 3). The RF rates at 3 months, 6 months, and 1, 3, and 5 years were 96.3%, 90.9%, 72.7%, 40.4%, and 33.3% (3- and 5-years’ RF were analyzed in 4 and 3 studies), respectively. The FFLR at 3, 6, and 12 months were 97.1%, 92.9, and 88.6%, respectively (FFLR was analyzed in 2 studies only). Overall, 40.7% of patients experienced progression, and 37.6% had hepatic progression. The OS rates at 3 months, 6 months, and 1, 3, and 5 years were 99.2%, 96.6%, 85.6%, 58.1%, and 43.3%, respectively.

**Figure 3:**
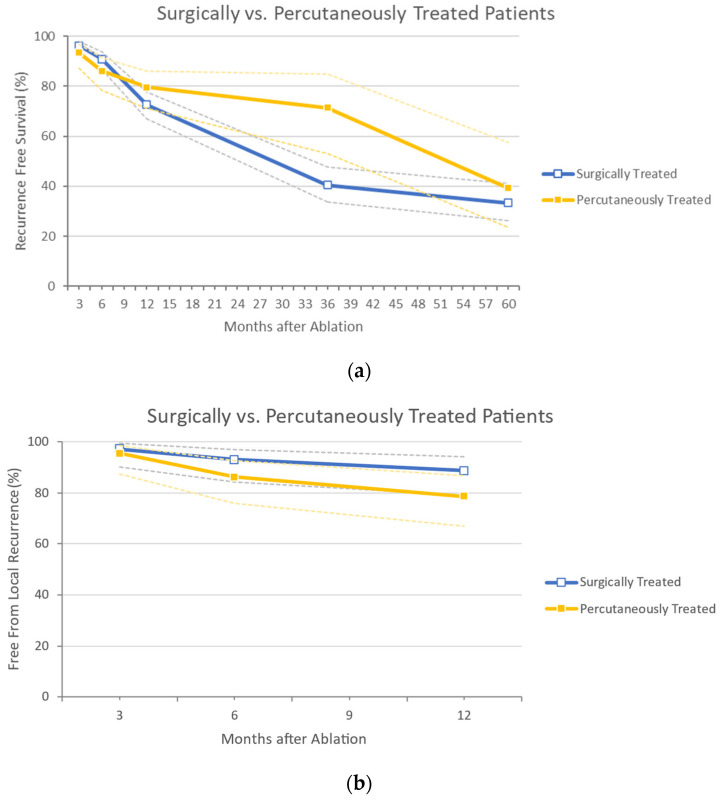

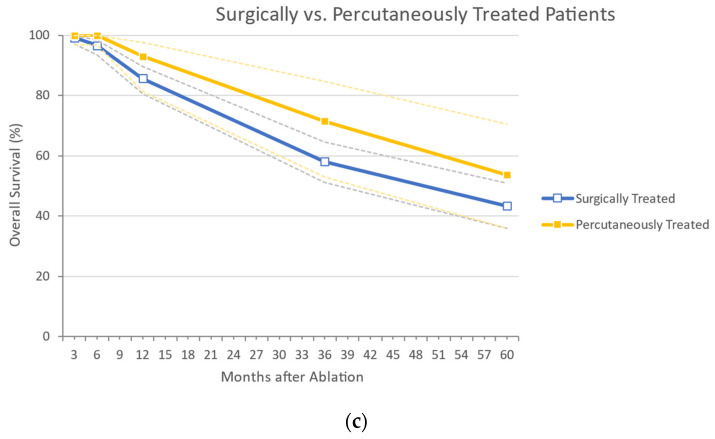
(**a**) Sub-analysis on studies comprising surgical vs. radiological approach, concerning Recurrence Free Survival outcomes. In dotted lines are indicated the 95% confidence intervals, (**b**) Sub-analysis on studies comprising surgical vs. radiological approach, concerning Free from Local Recurrence outcomes. In dotted lines are indicated the 95% confidence intervals, (**c**) Sub-analysis on studies comprising surgical vs. radiological approach, concerning Overall Survival outcomes. In dotted lines are indicated the 95% confidence intervals.

Only 4 studies report data concerning percutaneous MWA [31,33,34,36]. Overall, 98 lesions were treated in 108 patients (taking into account the missing data) [36]. The mean lesion diameter was 21.78 mm (±8.27 mm). Of all lesions, 73.5% were smaller than 30 mm. The majority of radiologically treated patients (80.6%) had a single lesion and had metachronous CRLM (76.8%). Percutaneous procedures encountered complications in 25.0% of patients, of which 8.5% were serious (CDs ≥ 3). The RF rates at 3 months, 6 months, and 1, 3, and 5 years were 93.5%, 86.1%, 79.6%, 71.4%, and 39.3%, respectively. The FFLR at 3, 6, and 12 months were 95.4%, 86.2%, and 78.5%, respectively. Overall, a recurrence was reported in 43.8% of patients, with a hepatic progression in 25.0%. The OS rates at 3 months, 6 months and 1, 3, and 5 years were 100%, 100%, 93.0%, 71.4%, and 53.6%, respectively. A graphic comparison between the two groups concerning RF, FFLR, and OS is depicted in Figure 3a–c.

#### Surgical MWA: Open vs. Laparoscopic Approach

Three studies analyzed the data concerning laparotomic surgery for MWA [30,35,37] (Table 10 and Table 11). In this group, 153 lesions were treated in 77 patients. The mean lesion diameter was 22.81 mm (±7.79 mm). All patients presented with more than one lesion. Open surgery resulted in complications for 37.7% of patients, of which 14.3% were severe (CDs ≥ 3). The RF rates at 3 and 12 months were 89.5% and 40.4%, respectively. The overall recurrence was 30.2%; the OS rates at 3 months, 6 months, and 1, 3, and 5 years were 100%, 98.7%, 81.8%, 42.9%, and 12.3%, respectively.

**Figure 4:**
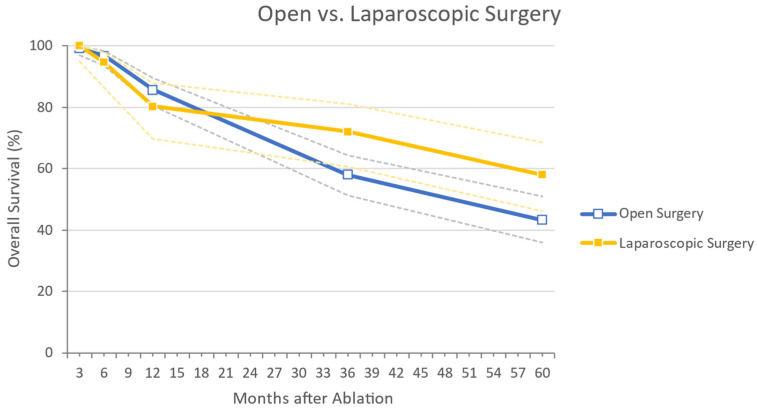
Sub-analysis on studies comprising lesions treated by open vs. laparoscopic surgical approach, concerning Overall Survival outcomes. In dotted lines are indicated the 95% confidence intervals.

Only 2 studies reported data on laparoscopic MWA [38,39]. In this group, 242 lesions were treated in 122 patients. The mean lesion diameter was 10.63 mm (±1.57 mm). The majority (73.0%) had more than one lesion. Laparoscopically treated patients showed a complication rate of 13.1%; data concerning complications with CDs ≥ 3 were available for one study only, and no further analysis was possible. The RF rates at 3, 6, and 12 months were 99.2%, 95.1%, and 85.5%, respectively; the overall recurrence rate was 44.3%. Data concerning OS rates were available for one study only, and no further analysis was possible. A graphic comparison between two groups concerning OS is depicted in Figure 4.

## 4. Discussion

Surgical resection with a parenchymal sparing technique is the gold standard of care for CRLM [40,41]. Unfortunately, for oncological reasons and due to the patients’ condition, few patients are candidates for curative-intent surgery. In this setting, when percutaneous thermal ablation techniques have been used in non-selected patients, oncological data from thermal ablation seemed to not be optimal when compared with a curative-intent surgical approach [32]. Modern CHT and target therapies for CRLM increased not only the resection rates but also the number of patients with complex CRLM and a high risk of recurrence in patients [32,40,42]. The main challenge is to define which patients can benefit most from surgery and thermal ablation techniques without increasing the morbidity and mortality rates [43].

Tabuse first developed the surgical technique of microwave coagulation in 1979 and applied it to the transection of hepatic parenchyma by coagulating the tumor tissue in many organs [44]. In the past, RFA and MWA have increased their usefulness in the context of CRLM treatment.

This study is the first systematic review focused only on the MWA of CRLM. In this review of 12 studies, we pooled 395 patients undergoing MWA for CRLM and reported the pooled analyses of OS, RF, and FFLR at 3 months, 6 months, and 1, 3, and 5 years.

A general observation of this systemic review was the performance of surgical MWA in 62.9% of patients. This underlines the need to explore other frontiers for CRLM treatment options, other than hepatic resection [20,29,30,32,35,37,38,39]. It was also reported that the complication rate was 26.8%, of which cases only 8.5% were severe (CDs ≥ 3). These data were in concordance with the literature data concerning outcomes after liver resection for CRLM [45]. The pooled analyses of the oncological outcome of MWA showed OS survival rates at 1, 3, and 5 years of 86.7%, 59.6%, and 44.8%, respectively. This was also comparable with OS rates reported after the curative liver resection of CRLM [45]. The RF rates at 1, 3, and 5 years after MWA were 65.1%, 44.6%, and 34.3%, respectively, which were better than the previously reported data [45].

The present results, as well as the reported literature data, show a common intention to treat CRLM that are less than 3 cm (91.6% of lesions) as a general principle of the advantageous influence of factors of the MWA [46,47]. In this subset of lesions, despite the presence of only 3 studies that analyzed oncological follow-up, 16.5% of patients experienced low-impact complications (CDs ≥ 3 = 0%). These results show a better safety profile than surgical resection and/or MWA from the Sweden Nationwide Registry, reporting severe complication rates of 16.4% and 7.0%, respectively [48].

Pooled analyses for RF rates and FFLR after MWA at 3, 6, and 12 months showed 91.8%, 83.5%, and 76.5% and 97.1%, 92.9%, and 88.6%, respectively, which was encouraging. In addition, hepatic progression rates of 32.4% and overall recurrence rates of 28.2% were in concordance with the data observed after resection. Interestingly, the OS rates after MWA for CRLM (100% at 3, 6, and 12 months) were encouraging and seemed better than surgical resection [48,49].

Analyzing the subgroup of surgical vs. radiological MWA approaches, no difference concerning complications was found. However, local hepatic control was more satisfactory after following the surgical approach, compared to radiological procedures. Indeed, the global RF and OS rates did not reflect the efficacy of treatment but, above all, the biological characteristics of CRLM. These results might be explained by the possible favorable tumor location for MWA and not for surgical resections, which are likely to be more complicated to treat in the same way as for MWA. As the rate of local recurrence seems dependent upon tumor location and its vessel proximity, in order to minimize the “heat sink effect”, pedicle clamping during MWA has been used successfully [20,32]. In terms of comparing surgical MWA, only the laparoscopic approach ensured a good RF rate. We believe that these results might be due to the fact that smaller-sized CRLM were treated using the laparoscopic approach (10 mm vs. 22.8 mm in an open approach) [50,51].

Despite our efforts to create a homogeneous comparison group, we acknowledge that our systematic review suffers from several limitations. Only 4 studies were prospective; in addition, some papers reported a heterogeneous number and size of ablated lesions, showing a general lack of standardization and follow-up protocols. Therefore, these results should be interpreted with caution, considering that most of them were extracted from clinically heterogeneous studies.

Finally, we believe that MWA represents a promising technique in the treatment of CRLM. When used appropriately, especially in selected patients with tumors less than 3 cm in size, the oncological results are promising. RFA and MWA are not mutually exclusive but they are additional, with the advantage of being able to perform MWA near the large blood vessels. For the bigger biliary ducts, the use of thermal ablation remains absolutely contraindicated. This extends the use of intraoperative MWA, which can be performed safely using a laparoscopy. Resection with or without MWA can achieve encouraging oncological results, with low morbidity allowing patients to receive their systemic drugs more quickly; thus, their care is not interrupted. We are also impatient to discover the final results of the ongoing prospective randomized COLLISION trial (Colorectal liver metastases: surgery versus thermal ablation trial) [52].

## 5. Conclusions

Our findings indicate that MWA could be a valid tool for CRLM treatment, especially for deep lesions and those smaller than 3 cm. The surgical approach for MWA could improve local control and reduce complications.

## Figures and Tables

**Table 1:** Inclusion and exclusion criteria. Abbreviations: colorectal liver metastases (CRLM), microwave ablation (MWA), chemotherapy (CHT), radiofrequency ablation (RFA).

Inclusion Criteria	Exclusion Criteria
MWA only	Combined procedures (i.e., RFA)
MWA + resections of other liver portions	MWA and subsequent resection of the same target
CRLM (+ mixed studies with identifiable results for CRLM only)	No adenocarcinoma CRLM
No lesions’ dimensional limits	Palliative treatment
Patients aged above 18 years old	Pregnant women
Sample > 10 Patients	Case reports and sample < 10 patients
Regardless neoadjuvant CT	
Recurrences	
Treated surgically and percutaneously	

**Table 2:** General characteristics of the included studies. The figure in curly brackets indicate the 95% confidence intervals.

Study ID	Year	Country	Study Type	Rand.	Study Design	Period	N-O QAS	Sample Size, *n*	CRLM Patients, *n*	MWA-Treated Patients, *n*	Ablations, *n*	Age, Mean ° (SD °)	Sex M/F
McEachron	2021	USA	Retrospective	No	Cohort	2009–2018	Fair	36	36	36	40 **	52 (±12.75)	21/15
Rhaiem	2020	France	Prospective	No	Cohort	November 2017– December 2018	Poor	19	19	19	23	67 (±10.5)	8/11
Takahashi	2018	USA	Retrospective	No	Case–Control	2014–2018	Good	105	105	51	121	NA	33/18
Shady	2017	USA	Retrospective	No	Case–Control	November 2019– April 2015	Good	110	110	48	72	NA	35/13
Yang	2017	China	Retrospective	No	Case–Control	January 2010– January 2016	Fair	179	179	71	121	51 (±5.33)	49/22
Song	2016	China	Retrospective	No	Case–Control	January 2012– January 2014	Good	62	62	28	NA	NA	15/13
Eng	2015	USA	Retrospective	No	Cohort	January 2009– April 2013	Poor	33	33	33	49	61 (±11.75)	24/9
Engstrand	2014	Sweden	Retrospective	No	Case–Control	October 2009– September 2012	Fair	81	81	20	7 * (4–22 *)	63.5 (±9.5)	9/11
Ierardi	2013	Italy	Prospective	No	Case–Control	May 2008– September 2011	Poor	25	17	17	23	65.75 (±7.75)	13/4
Stattner	2013	UK	Retrospective	No	Cohort	May 2005– December 2012	Good	43	43	43	95	64.5 (±11)	32/11
Shibata	2000	Japan	Prospective	Yes	Case–Control	December 1990– August 1887	Good	30	30	14	58	61.5 (±11.26)	8/6
Seki	1999	Japan	Prospective	No	Cohort	January 1994– May 1997	Poor	15	15	15	32	66 (±5.76)	10/5
Total	-	-	-	-	-	-		741	730	395	-	58.92 (±9.19)	257/138

Abbreviations—CRLM: colorectal liver metastases; MWA: microwave ablation; N-O QAS: Newcastle–Ottawa Quality Assessment Scale; Rand: randomization; SD: standard deviation; NA: missing data. *: median and range; **: intended not as MWA passages but as surgical procedures (re-treatment of 2 patients and 3 treatments on one patient); °: calculated using the Hozo and Wan method.

**Table 3:** Lesion characteristics. The figures in curly brackets indicate 95% confidence intervals.

Study ID	MWA-Treated Patients/ Lesions, *n*	Dimensions, Mean (SD), mm	Lesions < 30 mm, *n* (%)	Percutaneous/ Surgical Treatment, pt *n* (%)	Surgical Open/Lap, pt *n* (%)	Single/Multiple Lesions, pt *n* (%)	Synch, pt *n* (%)/ Meta, pt *n* (%)	Neoadj, *n* (%)/ Adj, *n* (%) Treatment
McEachron 2021	36/135	19 ° (±19.5 °)	132 (97.8%)	0/ 36 (100%)	13 * (32.5% *)/ 27 * (67.5% *)	NA/NA	13 (36.1%)/NA	36 (100%)/NA
Rhaiem 2020	19/23	15.25 ° (±2.75°)	23 (100%)	0/ 19 (100%)	15 (78.9%)/ 4 (21.1%)	15 (78.9%)/ 4 (21.1%)	8 (42.1%)/ 11 (57.9%)	17 (89%)/ 19 (100%)
Takahashi 2018	51/121	21.25 ° (±2.83 °)	121 (100%)	0/ 51 (100%)	0/ 51 (100%)	0/ 51 (100%)	NA/NA	28 (54.9%)/NA
Shady 2017	48/60	17 ° (±7.5 °)	56 (93.3%)	48 (100%)/ 0	0/0	40 (83.3%)/ 8 (17.7%)	NA/NA	NA/NA
Yang 2017	71/121	3 ° (±0.67°)	NA	0/ 71 (100%)	0/ 71 (100%)	33 (46.5%)/ 38 (53.5%)	NA/NA	NA/NA
Song 2016	28/NA	NA	NA	28 (100%)/ 0	0/0	18 (64.3%)/ 10 (35.7%)	10 (35.7%)/ 18 (64.3%)	NA/NA
Eng 2015	33/49	NA	42 (85.7%)	0/ 33 (100%)	NA/NA	NA/NA	NA/NA	32 (96.9%)/ 30 (90.9%)
Engstrand 2014	20/NA	NA	NA	0/ 20 (100%)	20 (100%)/ 0	0/ 20 (100%)	18 (90%)/ 2 (10%)	9 (45%)/ 13 (65%)
Ierardi 2013	17/23	34.52 ° (±13.25 °)	1 (4.8%)	17 (100%)/ 0	0/0	14 (82.4%)/ 3 (17.6%)	NA/NA	NA/17 (100%)
Stattner 2013	43/95	20.25 ° (±5.83 °)	NA	0/ 43 (100%)	43 (100%)/ 0	NA/NA	27 (62.8%)/ 16 (37.2%)	31 (72.1%)/NA
Shibata 2000	14/58	27 ° (±11 °)	NA	0/ 14 (100%)	14 (100%)/ 0	0/ 14 (100%)	NA/NA	NA/NA
Seki 1999	15/15	21.4 ° (±3.73 °)	15 (100%)	15 (100%)/ 0	0/0	15 (100%)/ 0	0/ 15 (100%)	0/ 15 (100%)
Total	395/700	17.62 ° (±7.87 °)	390 (91.6%) {88.5–93.8%}	108 (32.3%)/ 287 (85.9%) {27.5–37.5%}/ {81.8–89.2%}	92 (34.7%)/ 126 (47.5%) {29.2–40.6%}/ {41.6–53.5%}	135 (47.7%)/ 148 (52.3%) {41.9–53.5%}/ {46.5–58.1%}	76 (47.2%)/ 62 (49.6%) {39.7–54.9%}/ {40.9–58.3%}	153 (70.5%)/ 94 (90.4%) {64.1–76.2%}/ {83.2–94.7%}

Abbreviations—Lap: laparoscopic; SD: standard deviation; Synch: synchronous; Meta: metachronous; Neoadj: neoadjuvant; Adj: adjuvant; pt: calculated on the number of patients; mm: millimeters; NA: missing data. *: calculated on the number of surgical treatments (data excluded from further analysis); °: calculated using the Hozo method.

**Table 4:** The MWA apparatuses that were employed, with the ablation techniques and complications registered. The figures in curly brackets indicate 95% confidence intervals.

Study ID	Type of MWA Device	Type of MWA Needle	Ablation(s) Per Lesion	Average Ablation Time, min	Average Energy	Operation Time, min	Complica-tions *n* (%)	Complications Type, pt *n*	Clavien–Dindo ≥ 3, *n* (%)	Length of Stay, Days (Range)
McEachron 2021	NeuWaveTM Microwave Ablation System, Ethicon, Madison, WI, USA	Certus 140, 2.45 GHz ablation system, Certus PR XT (20 cm), or LK Max XT 25 cm probes (single, double or three probes: lesion cutoff 1.5–2.5 cm)	Single	NA	NA	NA	8 (22.2%)	Post-operative pain (3), tumor lysis syndrome (1)	1 (2.8%)	2.5 (0–28)
Rhaiem 2020	EmPrintTM Ablation System, Medtronic, Dublin, Ireland	2.45 GHz, 14 G probes with ThermosphereTM Technology	Single and multiple	5	75 W §	NA	6 (31.6%)	Evisceration (1), biliary fistula (1), peritonitis due to anastomotic leakage (1), heparin-induced thrombocytopenia (1), surgical site infection (1), MHV thrombosis (1)	0	NA
Takahashi 2018	EmPrintTM Ablation System, Covidien, Boulder, CO, USA	2.45 GHz, 14 Gauge antenna	NA	2.5–15	100 W	154 (±3 *)	7(13.7%)	NA	NA	(1–4)
Shady 2017	NeuWaveTM Microwave Ablation System; HS AMICATM; Microsulis (Angiodynamics, New York, NY, USA); and EmPrintTM Ablation System	NA	NA	NA	NA	NA	19 (39.6%)	PNX (11), hepatic artery- portal venous fistula (1), bowel perforation (1), bilomas (2), left portal vein thrombosis (1), sub-scapular hematoma (1), subcutaneous emphysema (1), pleural effusion (1)	6 (12.5%)	NA
Yang 2017	NA	NA	Single	70 (total)	NA	NA	9 (12.7%)	Perihepatic fluid collection (3), ascites (3), UTI (2), pleural effusion (1)	0	7 (5–19)
Song 2016	KY-2000 Microwave Ablation System (Kangyou Medical, Nanjing, Jiangsu, China)	2450 MHz antennae of three types (0.5, 0.7 and 1.1 cm tips Ø)	NA	NA	NA	NA	3 (10.7%)	Pain (3)	2 (7.1%)	5.9 (±0.9 *)
Eng 2015	ValleyLabTM Microwave Ablation Generator System, Covidien, Boulder, CO, USA	NA	NA	NA	NA	NA	18 (54.5%)	Intra-abdominal abscess drained radiologically (4), respiratory distress (3), biliary fistula (1)	8 (24.2%)	6 (1–32)
Engstrand 2014	Acculis^®^ Microwave Tissue Ablation System, Angiodynamics, Latham, NY, USA	NA	NA	NA	NA	235 (112–475)	12 (60%)	Multiple liver abscesses drained percutaneously (1), pleural effusion drained percutaneously (1), severe respiratory distress (3)	5 (25%)	10 (2–24)
Ierardi 2013	EvidentTM Microwave Ablation System, Covidien, USA	Straight 14.5-gauge antennas (12–17 cm of length), saline-perfused coaxial cable. Lesions of 3–4 cm Ø were treated with two antennas. Lesions of >4 cm Ø were treated with three antennas	Single	10	45 W	NA	4 (23.5%)	Abscess drained (1), pain (3), ascites (1)	1 (5.9%)	NA
Stattner 2013	Acculis^®^ Microwave Tissue Ablation System (Microsulis Medical Ltd., Dublin, UK)	2.65 GHz, shaft-cooled Accu2i pMTA antenna	NA	1.5	100 W	NA	15 (34.9%)	Re-intervention (3), death (1)	4 (9.3%)	7 (4–80)
Shibata 2000	Microwave tissue coagulator HSD-20M (Azwell, Osaka, Japan)	For coagulation of superficial tumors: 2-cm-long electrode (0.7 mm Ø; TM-20; Azwell). For deep tumors: 20-cm-long electrode (1.6 mm Ø; TMD- 16CBL; Azwell)	NA	2–20	60– 100 W	180 (±20 *)	2 (14.3%)	Hepatic abscess (1), bile duct fistula (1)	2 (14.3%)	NA
Seki 1999	Microtaze OT-110 M, Nippon Shoji, Azwell Inc., Osaka, Japan	MW electrode of 2.0 mm Ø, 25 cm length (MD-20 CDL- 10/25)	Multiple	1	80 W	NA	1 (6.7%)	Pleural effusion (1)	0	4
Total	-	-	-	-	-	184.16 ° (±26.45°)	104 (26.3%) {22.2–30.9%}	-	29 (8.4%) {5.9– 11.8%}	5.43 (NA)

Abbreviations—MW: microwave; PNX: pneumothorax; UTI: urinary tract infection; NA: missing data. Ø: diameter; *: mean and standard deviation; §: an additional treatment could be performed at 45 W for 3 min for nodules between 1.5 and 2 cm or 100 W for 1–3 min for larger nodules. °: means and standard deviations, calculated using the Hozo method on the complete available data (conversions for each study are not shown).

**Table 5:** Survival analysis. Figures in curly brackets indicate the 95% confidence intervals.

Study ID	Follow-Up Mean° (SD°), Months	3 Months RF	6 Months RF	1 Year RF	3 Years RF	5 Years RF	Total RF	3 Months FFLR	6 Months FFLR	1 Year FFLR
McEachron 2021	NA	100%	100%	100%	54%	54%	50%	NA	NA	NA
Rhaiem 2020	11.75 (±2.25)	90.3%	61.9%	47.6%	NA	NA	NA	94.7%	84.2%	78.9%
Takahashi 2018	17 (±2.25)	97.7%	95.8%	91.5%	NA	NA	NA	97.7%	95.8%	91.5%
Shady 2017	NA	97%	85.8%	79%	NA	NA	62%	97%	85.8%	79%
Yang 2017	NA	100%	94.3%	80.3%	56.2%	39%	39%	NA	NA	NA
Song 2016	NA	100%	100%	96.7%	71.4%	39.3%	10%	NA	NA	NA
Eng 2015	17.46 (±11.51)	96.9%	87.9%	66.7%	19.3%	NA	19.3%	NA	NA	NA
Engstrand 2014	28.25 (±11.25)	NA	NA	NA	NA	NA	25%	NA	NA	NA
Ierardi 2013	15.77 (±8.25)	88.2%	82.3%	70.6%	NA	NA	64.7%	88.2%	88.2%	76.5%
Stattner 2013	29.25 (±20.25)	85.7%	85.7%	31%	22%	6%	27.9%	NA	NA	NA
Shibata 2000	NA	100%	NA	70%	NA	NA	11.3%	NA	NA	NA
Seki 1999	21 (±8.09)	73.4%	66.7%	60%	NA	NA	NA	NA	NA	NA
Total	20.57 ° (±9.57°)	95.5% {92.9– 97.2%}	89.5% {85.9– 92.2%}	65.1% {60.1– 69.7%}	44.6% {38– 51.3%}	34.3% {27.7– 41.5%}	37.1% {31.9– 42.6%}	96.3% {91.6– 98.4%}	89.6% {83.3– 93.7%}	83.7% {76.6– 88.9%}
**Study** **ID**	**Hepatic** **Progression** **,** **pt** ***n* (%)**	**Extra-Hepatic Progression,** **pt *n* (%)**		**Overall** **Recurrence,** **pt *n* (%)**		**3** **Months** **OS**	**6** **Months** **OS**	**1** **Year** **OS**	**3** **Years** **OS**	**5** **Years** **OS**
McEachron 2021	18 (50%)	1 (2.8%)		18 (50%)		100%	100%	100%	75%	63%
Rhaiem 2020	7 (36.8%)	NA		7 (36.8%)		100%	100%	100%	NA	NA
Takahashi 2018	NA	NA		11 (21.6%)		NA	NA	NA	NA	NA
Shady 2017	NA	NA		23 (47.9%)		NA	NA	NA	NA	NA
Yang 2017	NA	NA		43 (60.6%)		100%	94.5%	80.2%	72%	58%
Song 2016	NA	NA		NA		100%	100%	89.1%	71.4%	53.6%
Eng 2015	10 (30.3%)	12 (36.4%)		13 (39.4%)		93.9%	90.9%	81.8%	45.7%	NA
Engstrand 2014	17 (85%)	11 (55%)		15 (75%)		100%	100%	90%	41.5%	NA
Ierardi 2013	4 (23.5%)	2 (11.8%)		6 (35.3%)		100%	100%	NA	NA	NA
Stattner 2013	5 (11.6%)	22 (51.2%)		4 (9.3%)		100%	100%	82%	40%	12%
Shibata 2000	NA	NA		NA		100%	95%	71%	57%	14%
Seki 1999	4 (26.6%)	6 (40%)		6 (40%)		100%	100%	100%	NA	NA
Total	65 (35.5%) {28.9–42.7%}	54 (32.9%) {26.2–40.4%}		146 (41.36%) {36.3–46.6%}		99.3% {97.6– 99.8%}	97.3% {94.8– 98.6%}	86.7% {82.2– 90.2%}	59.6% {53.3– 65.5%}	44.8% {37.9– 51.8%}

Abbreviations—RF: recurrence-free; FFLR: free from local recurrence; SD: standard deviation; NA: missing data. °: means and standard deviations calculated using the Hozo method on complete available data. OS: overall survival; DFS: disease-free survival; pt.: patients; NA: missing data.

**Table 6:** Sub-analysis on studies comprising lesions of <30 mm only: general data. Figures in curly brackets indicate 95% confidence intervals.

Study ID	Patients/ Lesions Treated, *n*	Dimensi-ons, Mean (SD), mm	Percutaneous/ Surgical Treatment, pt *n* (%)	Surgical Open/Lap, pt *n* (%)	Single/ Multiple Lesions, pt *n* (%)	Synch, pt *n* (%)/Meta, pt *n* (%)	Neoadj, *n* (%) ±/ Adj, *n* (%) Treatment	Complica-tions *n* (% pt.)	CDs ≥3, *n* (%)
Rhaiem 2020	19/23	15.25 ° (±2.75 °)	0±/ 19 (100%)	15 (78.9%) ±/ 4 (21.1%)	15 (78.9%) ±/ 4 (21.1%)	8 (42.1%) ±/ 11 (57.9%)	17 (89%) ±/ 19 (100%)	6 (38%)	0
Takahashi 2018	51/121	21.25 ° (±2.83 °)	0±/ 51 (100%)	0±/ 51 (100%)	0±/ 51 (100%)	NA/NA	28 (54.9%)/NA	7 (13.7%)	NA
Seki 1999	15/15	21.4 (±3.73 °)	15 (100%) ±/ 0	0/0	15 (100%) ±/ 0	0±/ 15 (100%)	0±/ 15 (100%)	1 (6.7%)	0
Total	85/159	20.39° (±2.90°)	15 (17.6%)/ 70 (82.4%) {11–27.1%} ±/ {72.3–89%}	15 (17.7%)/ 55 (64.7%) {11–27.1%} ±/ {54.1–74%}	30 (35.3%)/ 55 (64.7%) {26.9–45.9%} ±/ {54.1–74%}	8 (23.5%)/ 26 (76.5%) {12.4–40%} ±/ {60–87.6%}	45 (52.9%)/ 34 (100%) {42.4–63.2%} ±/ {89.9–100%}	14 (16.5%) {10.1–25.8%}	0 (0%) {0–10.2%}

Abbreviations—SD: standard deviation; Lap: laparoscopic; Synch: synchronous; Meta: metachronous; Neoadj: neoadjuvant; Adj: adjuvant; CDs: Clavien–Dindo score; pt: calculated on the number of patients; mm: millimeters; NA: missing data. °: calculated using the Hozo method.

**Table 7:** Sub-analysis on studies comprising lesions of <30 mm only: oncological data. Figures in curly brackets indicate 95% confidence intervals.

Study ID	3 Months RF	6 Months RF	1 Year RF	3 Months FFLR	6 Months FFLR	1 Year FFLR	Hepatic Progression, pt *n* (%)	Overall Recurrence, pt *n* (%)	3 Months OS	6 Months OS	1 Year OS
Rhaiem 2020	90.3%	61.9%	47.6%	94.7%	84.2%	78.9%	7 (36.84%)	7 (36.8%)	100%	100%	100%
Takahashi 2018	97.7%	95.8%	91.5%	97.7%	95.8%	91.5%	NA	11 (21.6%)	NA	NA	NA
Seki 1999	73.4%	66.7%	60%	NA	NA	NA	4 (26.6%)	6 (40%)	100%	100%	100%
Total	91.8% {83.9– 95.9%}	83.5% {74.2– 89.9%}	76.5% {66.4– 84.2%}	97.1% {90.2– 99.2%}	92.9% {84.3– 96.9%}	88.6% {79– 94.1%}	11 (32.4%) {19.1–49.1%}	24 (28.2%) {19.8–38.6%}	100% {89.9– 100%}	100% {89.9– 100%}	100% {89.9– 100%}

Abbreviations—RF: recurrence-free; FFLR: free from local recurrence; OS: overall survival; pt: calculated on the number of patients; NA: missing data.

**Table 8:** Sub-analysis on studies comprising a surgical vs. a radiological approach. Figures in curly brackets indicate 95% confidence intervals.

	Study ID	Patients/ Lesions Treated, *n*	Dimensions, Mean (SD), mm	Lesions <30 mm, *n* (%)	Surgical Open/Lap, pt *n* (%)	Single/Multiple Lesions, pt *n* (%)	Synch, pt *n* (%)/Meta, pt *n* (%)	Complications *n* (%)	CDs ≥3, *n* (%)
**Surgical Approach**	McEachron 2021	36/135	19 ° (±19.5 °)	132 (97.78%)	13* (32.5% *)/ 27* (67.5% *)	NA	13 (36.11%)/NA	8 (22.2%)	1 (2.8%)
	Rhaiem 2020	19/23	15.25 ° (±2.75 °)	23 (100%)	15 (78.95%)/ 4 (21.05%)	15 (78.95%)/ 4 (21.05%)	8 (42,1%)/ 11 (57.89%)	6 (38%)	0
	Takahashi 2018	51/121	21.25 ° (±2.83 °)	121 (100%)	0/ 51 (100%)	0/ 51 (100%)	NA/NA	7 (13.7%)	NA
	Yang 2017	71/121	3 ° (±0.67°)	NA	0/ 71 (100%)	33 (46.48%)/ 38 (53.52%)	NA/NA	9 (12.7%)	0
	Eng 2015	33/49	NA	42 (85.71%)	NA	NA	NA/NA	18 (54.5%)	8 (24.2%)
	Engstrand 2014	20/NA	NA	NA	20 (100%)/ 0	0/ 20 (100%)	18 (90%)/ 2 (10%)	12 (60%)	5 (25%)
	Stattner 2013	43/95	20.25 ° (±5.83°)	NA	43 (100%)/ 0	NA	27 (62.8%)/ 16 (37.2%)	15	4
	Shibata 2000	14/58	27 (±11)	NA	14 (100%)/ 0	0/ 14 (100%)	NA/NA	2	2
	Total	287/602	15.51 ° (±7.16°)	318 (96.9%) {94.5–98.3%}	92 (42.2%)/ 126 (57.8%) {35.8–48.8%}/ {51.2–64.2%}	48 (27.4%)/ 127 (72.6%) {21.4–34.5%}/ {65.5–78.6%}	66 (55.9%)/ 29 (35.4%) {46.9–64.7%}/ {25.9–46.2%}	77 (26.8%) {22–32.2%}	20 (8.5%) {5.6–12.7%}
**Radiological Approach**	Shady 2017	48/60	17 ° (±7.5 °)	56 (93.33%)	−	40 (83.3%)/ 8 (17.67%)	NA/NA	19	6 (12%)
	Song 2016	28/NA	NA	NA	−	18 (64.3%)/ 10 (35.7%)	10 (35.7%)/ 18 (64.3%)	3 (10.7%)	2
	Ierardi 2013	17/23	34.52 (±13.25 °)	1 (4.8%)	−	14 (82.4%)/ 3 (17.6%)	NA/NA	4 (23.5%)	1 (5.9%)
	Seki 1999	15/15	21.4 (±3.73 °)	15 (100%)	−	15 (100%)/ 0	0/ 15 (100%)	1 (6.7%)	0
	Total	108/98	21.78 ° (±8.27°)	72 (73.5%) {63.9–81.2%}	−	87 (80.6%)/ 21 (19.4%) {72.1–86.9%}/ {13.1–27.9%}	10 (23.3%)/ 33 (76.8%) {13.2–37.7%}/ {62.3–86.9%}	27 (25%) {17.8–33.9%}	9 (8.3%) {4.5–15.1%}

Abbreviations—SD: standard deviation; Lap: laparoscopic; Synch: synchronous; Meta: metachronous; CDs: Clavien-Dindo score; pt: calculated on the number of patients; mm: millimeters; NA: missing data. *: calculated on the number of surgical treatments (data excluded from further analysis); °: calculated using the Hozo method.

**Table 9:** Sub-analysis on studies comprising a surgical vs. a radiological approach, concerning oncological outcomes. Figures in curly brackets indicate 95% confidence intervals.

	Study ID	3 Months RF	6 Months RF	1 Year RF	3 Years RF	5 Years RF	3 Months FFLR	6 Months FFLR	1 year FFLR	Hepatic Progression pt *n* (%)	Overall Recurrence, pt *n* (%)	3 Months OS	6 Months OS	1 Year OS	3 Years OS	5 Years OS
**Surgical Approach**	McEachron 2021	100%	100%	100%	54%	54%	NA	NA	NA	18 (50%)	18 (50%)	100%	100%	100%	75%	63%
	Rhaiem 2020	90.3%	61.9%	47.6%	NA	NA	94.7%	84.2%	78.9%	7 (36.8%)	7 (36.8%)	100%	100%	100%	NA	NA
	Takahashi 2018	97.7%	95.8%	91.5%	NA	NA	97.7%	95.8%	91.5%	NA	11 (21.6%)	NA	NA	NA	NA	NA
	Yang 2017	100%	94.3%	80.3%	56.2%	39%	NA	NA	NA	NA	43 (60.6%)	100%	94.5%	80.2%	72%	58%
	Eng 2015	96.9%	87.9%	66.7%	19.3%	NA	NA	NA	NA	10 (30.3%)	13 (39.4%)	93.9%	90.9%	81.8%	45.7%	NA
	Engstrand 2014	NA	NA	NA	NA	NA	NA	NA	NA	17 (85%)	15 (75%)	100%	100%	90%	41.5%	NA
	Stattner 2013	85.7%	85.7%	31%	22%	6%	NA	NA	NA	5 (11.63%)	4 (9.3%)	100%	100%	82%	40%	12%
	Shibata 2000	100%	NA	70%	NA	NA	NA	NA	NA	NA	NA	100%	95%	71%	57%	14%
	Total	96.3% {93.2– 97.9%}	90.9% {86.7– 93.9%}	72.7% {67– 77.7%}	40.4% {33.6– 47.7%}	33.3% {26.3– 41.2%}	97.1% {90.2– 99.2%}	92.9% {84.3– 96.9%}	88.6% {79– 94.1%}	57 (37.6%) {30.4–45.7%}	111 (40.7%) {35–46.6%}	99.2% {96.9– 99.7%}	96.6% {93.5– 98.3%}	85.6% {80.54– 89.50%}	58.1% {51.1– 64.4%}	43.3% {35.9– 50.9%}
**Radiological** **Approach**	Shady 2017	97%	85.8%	79%	NA	NA	97%	85.8%	79%	NA	23 (47.9%)	NA	NA	NA	NA	NA
	Song 2016	100%	100%	96.7%	71.4%	39.3%	NA	NA	NA	NA	NA	100%	100%	89.1%	71.4%	53.6%
	Ierardi 2013	88.2%	82.4%	70.6%	NA	NA	88.2%	88.2%	76.5%	4 (23.5%)	6 (35.3%)	100%	100%	NA	NA	NA
	Seki 1999	73.4%	66.7%	60%	NA	NA	NA	NA	NA	4 (26.6%)	6 (40%)	100%	100%	100%	NA	NA
	Total	93.5% {87.2– 96.8%}	86.1% {78.3– 91.4%}	79.6% {71.1– 86.2%}	71.4% {52.9– 84.8%}	39.3% {23.6– 57.6%}	95.4% {87.3– 98.4%}	86.2% {75.7– 92.5%}	(78.5%) {67–86.7%}	8 (25%) {13.3–42.1%}	35 (43.8%) {33.4–54.7%}	100% {93.9– 100%}	100% {93.9– 100%}	93% {81.4– 97.6%}	71.4% {52.9– 84.8%}	53.6% {35.8– 70.5%}

Abbreviations—RF: recurrence-free; FFLR: free from local recurrence; OS: overall survival; pt: calculated on the number of patients; NA: missing data.

**Table 10:** Sub-analysis on studies comprising lesions treated by open vs. laparoscopic surgical approach: general data. Figures in curly brackets indicate 95% confidence intervals.

	Study ID	Patients/ Lesions Treated, *n*	Dimensions, Mean (SD), mm	Single/Multiple Lesions, pt *n* (%)	Complications, *n* (%)	CDs ≥ 3, *n* (%)
**Open**	Engstrand 2014	20/NA	NA	0/ 20 (100%)	12 (60%)	5 (25%)
	Stattner 2013	43/95	20.25 ° (±5.83 °)	NA	15	4
	Shibata 2000	14/58	27 (±11)	0/ 14 (100%)	2	2
	Total	77/153	22.81 ° (±7.79 °)	0 (0%)/34 (100%) {0–10.2%}/{89.9–100%}	29 (37.7%) {27.7–48.8%}	11 (14.3%) {8.2–23.8%}
**Laparoscopic**	Takahashi 2018	51/121	21.25 ° (±2.83 °)	0 51 (100%)	7 (13.7%)	NA
	Yang 2017	71/121	3 ° (±0.67 °)	33 (46.5%)/ 38 (53.5%)	9 (12.7%)	0
	Total	122/242	10.63 ° (±1.57 °)	33 (27.1%)/89 (73%) {19.9–35.5%}/{64.5–80%}	16 (13.1%) {8.2–20.3%}	-

Figures in squared brackets indicated 95% confidence intervals. Abbreviations—SD: standard deviation; CDs: Clavien–Dindo score; pt: calculated on the number of patients; mm: millimeters; NA: missing data. °: calculated using the Hozo method using complete available data.

**Table 11:** Sub-analysis on studies comprising lesions treated by an open vs. a laparoscopic surgical approach: oncological outcomes. Figures in curly brackets indicate 95% confidence intervals.

	Study ID	3 Months RF	6 Months RF	1 Year RF	Overall Recurrence, pt *n* (%)	3 Months OS	6 Months OS	1 Year OS	3 Years OS	5 Years OS
**Open**	Engstrand 2014	NA	NA	NA	15 (75%)	100%	100%	90%	41.5%	NA
	Stattner 2013	85.7%	85.7%	31%	4 (9.3%)	100%	100%	82%	40%	12%
	Shibata 2000	100%	NA	70%	NA	100%	95%	71%	57%	14%
	Total	89.5% {78.9– 95.1%}	-	40.4% {28.6– 53.3%}	19 (30.2%) {20.2–42.4%}	100% {95.3– 100%}	98.7% {93– 99.8%}	81.8% {71.8– 88.9%}	42.9% {32.4– 54%}	12.3% {6.1– 23.3%}
**Laparoscopic**	Takahashi 2018	97.7%	95.8%	91.5%	11 (21.6%)	NA	NA	NA	NA	NA
	Yang 2017	100%	94.3%	80.3%	43 (60.6%)	100%	94.5%	80.2%	72%	58%
	Total	99.2% {95.5– 99.9%}	95.1% {89.7– 97.7%}	85.5% {77.9– 90.5%}	54 (44.3%) {35.8–53.1%}	-	-	-	-	-

Abbreviations—RF: recurrence-free; OS: overall survival; pt: calculated on the number of patients; NA: missing data.
